# The Dynamic Chemistry of Orthoesters and Trialkoxysilanes: Making Supramolecular Hosts Adaptive, Fluxional, and Degradable

**DOI:** 10.1021/acs.accounts.3c00738

**Published:** 2024-01-29

**Authors:** Selina Hollstein, Max von Delius

**Affiliations:** Institute of Organic Chemistry, Ulm University, Albert-Einstein-Allee 11, 89081 Ulm, Germany

## Abstract

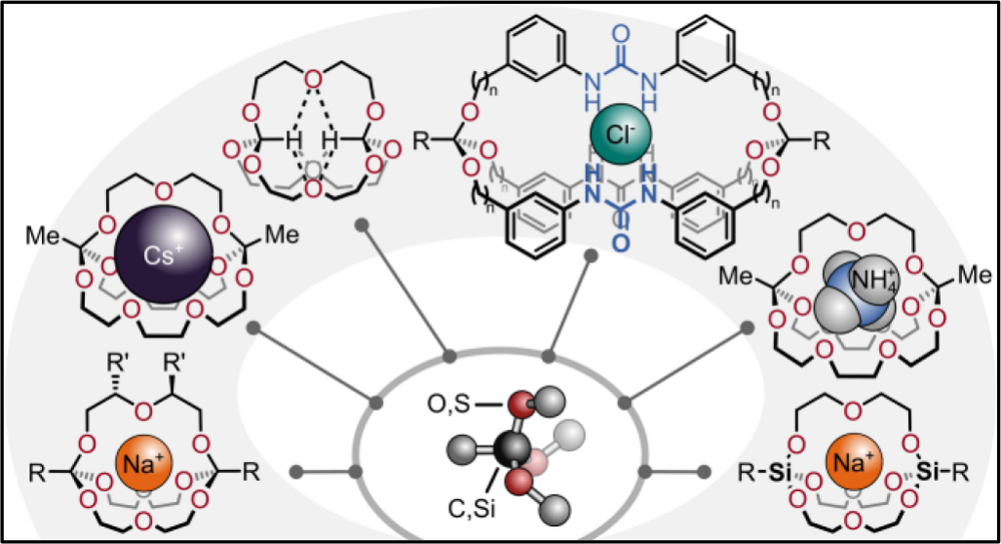

The encapsulation of ions into macro(bi)cyclic hosts lies at the core of supramolecular chemistry. While chemically inert hosts such as crown ethers (synthesis) and cyclodextrins (Febreze) have enabled real-world applications, there is a wider and accelerating trend toward functional molecules and materials that are stimuli-responsive, degradable, or recyclable. To endow supramolecular hosts with these properties, a deviation from ether C–O bonds is required, and functional groups that engage in equilibrium reactions under relatively mild conditions are needed.

In this Account, we describe our group’s work on supramolecular hosts that comprise orthoester and trialkoxysilane bridgeheads. In their simplest structural realization, these compounds resemble both Cram’s crown ethers (macrocycles with oxygen donor atoms) and Lehn’s cryptands (macrobicycles with 3-fold symmetry). It is therefore not surprising that these new hosts were found to have a natural propensity to bind cations relatively strongly. In recent work, we were also able to create anion-binding hosts by placing disubstituted urea motifs at the center of the tripodal architecture. Structural modifications of either the terminal substituents (e.g., H vs CH_3_ on the bridgehead), the diol (e.g., chiral), or the bridgehead atom itself (Si vs C) were found to have profound implications on the guest-binding properties.

What makes orthoester/trialkoxysilane hosts truly unique is their dynamic covalent chemistry. The ability to conduct exchange reactions with alcohols at the bridgehead carbon or silicon atom is first and foremost an opportunity to develop highly efficient syntheses. Indeed, all hosts presented in this Account were prepared via templated self-assembly in yields of up to 90%. This efficiency is remarkable because the macrobicyclic architecture is established in one single step from at least five components. A second opportunity presented by dynamic bridgeheads is that suitable mixtures of orthoester hosts or their subcomponents can be adaptive, i.e. they respond to the presence of guests such that the addition of a certain guest can dictate the formation of a preferred host. In an extreme example of dynamic adaptivity, we found that ammonium ions can fulfill the dual role of catalyst for orthoester exchange and cationic template for efficient host formation, representing an unprecedented example of a fluxional supramolecular complex. The third implication of dynamic bridgeheads is due to the reaction of orthoesters and trialkoxysilanes with water instead of alcohols. We describe in detail how the hydrolysis rate differs strongly between *O*,*O*,*O*-orthoesters, *S*,*S*,*S*-trithioorthoesters, and trialkoxysilanes and how it is tunable by the choice of substituents and pH.

We expect that the fundamental insights into exchange and degradation kinetics described in this Account will be useful far beyond supramolecular chemistry.

## Key References

- BrachvogelR. C.; HampelF.; von DeliusM.Self-Assembly of Dynamic Orthoester Cryptates. Nat. Commun.2015, 6, 712925997913 10.1038/ncomms8129PMC4455094.^1^ In this paper, we report the first example of the dynamic covalent self-assembly of a monometallic cryptate using orthoesters as acid-labile bridgeheads.
- WangX.; ShyshovO.; HanževačkiM.; JägerC. M.; von DeliusM.Ammonium Complexes of Orthoester Cryptands Are Inherently Dynamic and Adaptive. J. Am. Chem. Soc.2019, 141, 8868–887631117548 10.1021/jacs.9b01350.^2^ We demonstrate that inherently dynamic host–guest complexes are obtained when orthoester cryptates are exposed to ammonium ions that simultaneously act as acid catalysts and templates via H bonding.
- HollsteinS.; ShyshovO.; HanževačkiM.; ZhaoJ.; RudolfT.; JägerC. M.; von DeliusM.Dynamic Covalent Self-Assembly of Chloride- and Ion-Pair-Templated Cryptates. Angew. Chem., Int. Ed.2022, 61, e20220183110.1002/anie.202201831PMC940085135384202.^3^ We show that cryptates assembled from orthoester bridgeheads and urea-diols are capable of encapsulating biologically relevant anions, as well as cesium chloride.
- HollsteinS.; ErdmannP.; UlmerA.; LöwH.; GrebL.; von DeliusM.Trialkoxysilane Exchange: Scope, Mechanism, Cryptates and pH-Response. Angew. Chem., Int. Ed.2023, 62, e20230408310.1002/anie.20230408337114678.^4^ We report a detailed understanding of trialkoxysilane exchange and the first example of a self-assembled trialkoxysilane host–guest complex.

## Introduction

Dynamic covalent chemistry (DCvC) combines the dynamic features of supramolecular chemistry with the robustness of covalent bonds, thereby enabling self-assembly processes with error correction. Popular exchange reactions in this area include imine, disulfide, hydrazone, (thio)ester, and boronic ester exchange.^5,6^ The unique feature of DCvC, i.e., the reversibility of the underlying transformations, makes it a powerful tool for identifying ligands for medicinally relevant biotargets^7,8^ or the synthesis of functional materials such as recyclable polymers,^9,10^ shape-persistent porous cages,^11,12^ and porous framework materials.^13,14^ The use of DCvC in supramolecular host–guest chemistry has recently attracted considerable interest.^15,16^ Representative examples for additions to the toolbox of DCvC include tetrazines,^17^ triazines,^18^ (hemi)aminals,^19^ amidines,^20^ imides,^21^ (cyclo)benzoins,^22^ carbamates,^23^ ureas,^24^ dithioacetals,^25^ diselenides,^26^ alkynes,^27^ azines,^28^ and even amides.^29^

**Figure 1:**
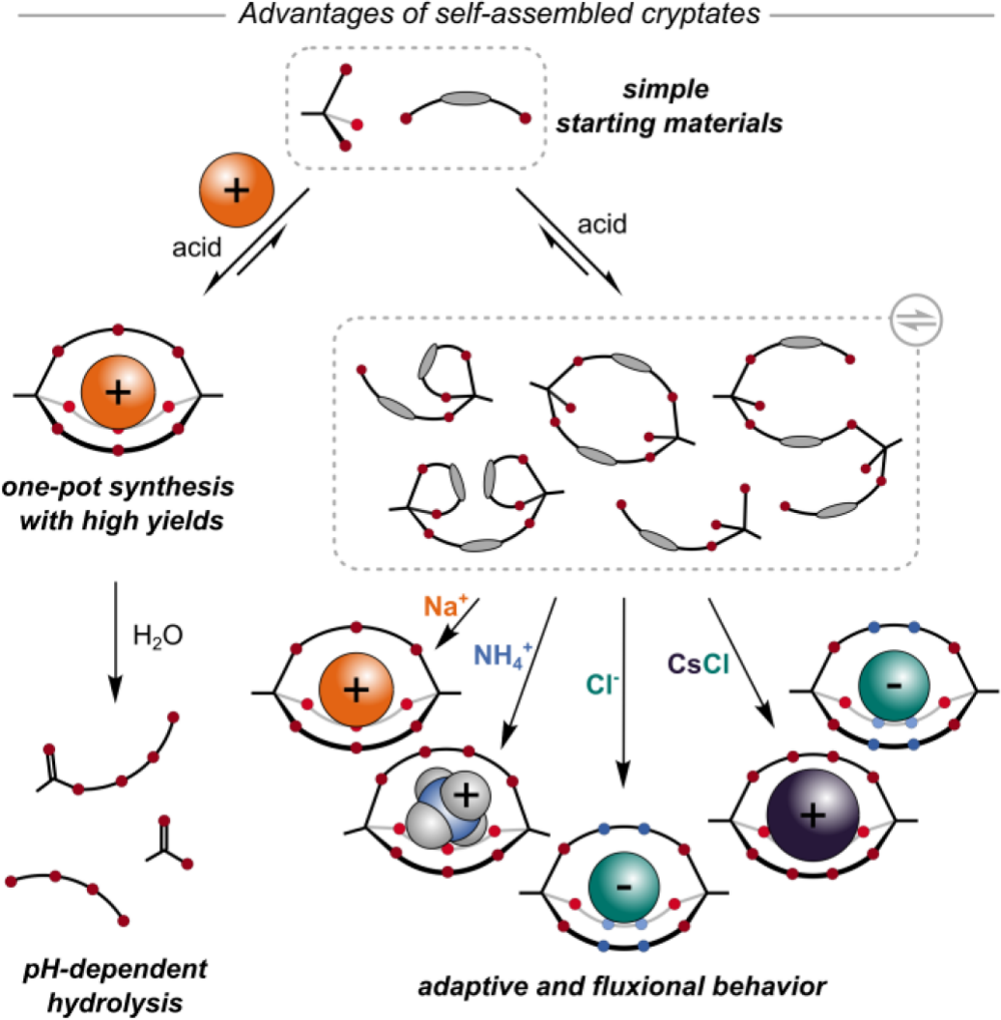
Appealing properties of orthoester/trialkoxysilane hosts.

Our group recently explored the exchange reactions of simple (trithio)orthoesters and trialkoxysilanes with alcohols/thiols. These reactions are related to established DCvC motifs of (thiol-)thioester and (alcohol-)acetal exchange.^30^ The tripodal geometries of the orthoester and trialkoxysilane motifs opened the opportunity for the self-assembly of small orthoester/trialkoxysilane cryptates, which exhibit appealing properties (Figure 1). In this Account, we summarize, discuss, and contextualize this body of work.

## Self-Assembly of Metal-Templated Orthoester Cryptates

The exchange of orthoesters with alcohols has long been known as a useful method in synthesis and catalysis,^31^ yet it was typically performed under rather harsh conditions.^32^ Motivated to make this method useful in the context of supramolecular chemistry, we set out to study the reaction and its scope at room temperature and in dilute solution.^33^ A notable peculiarity of orthoesters is that they hydrolyze irreversibly to the corresponding esters in an acidic aqueous medium. For this reason, orthoester exchange has to be carried out in anhydrous solvents, and any reagents should be dried (either before use or in situ by addition of an excess of “sacrificial” orthoester). This greatest weakness of orthoesters is however also their greatest strength, as their strongly pH-dependent hydrolysis provides an excellent opportunity to create degradable mimics of polyethylene^34^ or systems for ocular drug delivery.^35^

**Figure 2:**
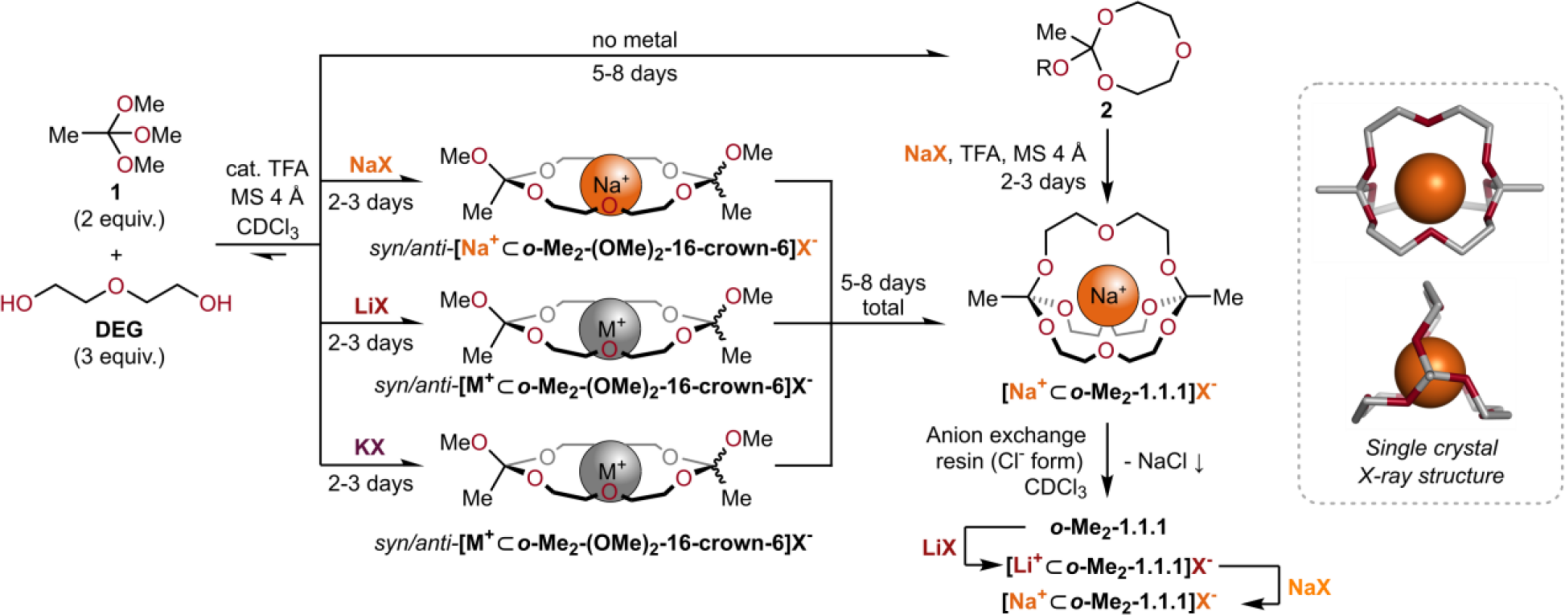
Self-assembly of orthoester cryptates and their complex behavior. Without a metal template, eight-membered ring **2** is formed. Addition of Na^+^, Li^+^, and K^+^ salts leads to the formation of crown ether intermediates. All macrocyclic products can be converted to cryptate **[Na**^**+**^**⊂o-Me**_**2**_**-1.1.1]** within ca. 1 week upon addition of sodium ions. The template can be removed by an anion exchange resin, and the empty cryptand can subsequently be converted to the lithium cage **[Li**^**+**^**⊂o-Me**_**2**_**-1.1.1]**. Right: single crystal X-ray structure of **[Na**^**+**^**⊂o-Me**_**2**_**-1.1.1]**.^1^

Having identified mild reaction conditions (e.g., 0.1 mol % trifluoroacetic (TFA)) and having explored simple metal template effects,^33^ we envisioned that orthoesters could form the two bridgeheads in macrobicyclic compounds that bear a strong structural similarity to Lehn’s iconic aza-cryptands published first in 1969.^36^ To our delight, we found that the two bulk chemicals, trimethyl orthoacetate (**1**) and di(ethylene glycol) (**DEG**), could be transformed into such a cryptate, provided that a suitable sodium salt was added as a template, which is necessary because these small cages are not shape-persistent (Figure 2). The addition of molecular sieves (MS) to the reaction mixture was highly fortuitous. We had originally meant to keep the medium anhydrous in this way, but we later learned that MS assists with the self-assembly reaction by providing a thermodynamic sink for the released methanol and thereby shifts the equilibrium toward the product. We were able to characterize the cryptate **[Na**^**+**^**⊂*****o*****-Me**_**2**_**-1.1.1]BArF**^**–**^ (where BArF^–^ = tetrakis[3,5-bis(trifluoromethyl)phenyl]borate) by single crystal X-ray crystallography (SCXRD, Figure 2)^1^ and were pleased, when others took this work as an inspiration to self-assemble related architectures using imine exchange.^15,37^

To understand the effect of the metal template, we performed the self-assembly reaction in the absence of any metal salt and in the presence of other metal templates (Figure 2). Without any template, we mainly observed the formation of the 8-membered cyclic product **2**, which can be converted to cryptate **[Na**^**+**^**⊂*****o*****-Me**_**2**_**-1.1.1]BArF**^**–**^ upon addition of NaBArF. In subsequent experiments, we used LiTPFPB (lithium tetrakis(pentafluorophenyl)borate), NaBArF, or KBArF as metal templates. In all cases, the formation of orthoester crown ethers ***o*****-Me**_**2**_**-(OMe)**_**2**_**-16-crown-6** in a mixture of *syn* and *anti* diastereomers (1:1) was observed before the dynamic system mysteriously converged to the sodium-encapsulating cryptate **[Na**^**+**^**⊂*****o*****-Me**_**2**_**-1.1.1]** irrespective of the salt added at the beginning of the experiment. We quickly realized that MS 4 Å (zeolite type A) were capable of exchanging Na^+^ with the metal ion (e.g., Li^+^, K^+^, Ag^+^) in the added salts, so the apparent mystery was revealed as a case of LeChatelier’s principle.

Despite this evident selectivity of the cryptand for a sodium guest, we managed to remove the guest from **[Na**^**+**^**⊂*****o*****-Me**_**2**_**-1.1.1]** by treatment with an anion exchange resin (Lewatit MP-64). This allowed us to isolate the parent cryptand ***o*****-Me**_**2**_**-1.1.1** and turn it into the lithium cryptate **[Li**^**+**^**⊂*****o*****-Me**_**2**_**-1.1.1]TPFPB**^**–**^ by the addition of the corresponding lithium salt. Addition of NaBArF to this complex led to **[Na**^**+**^**⊂*****o*****-Me**_**2**_**-1.1.1]BArF**^**–**^, again confirming the preference for sodium (Figure 2).^1^

In a comprehensive follow-up study published in 2018, we explored the scope of metal-templated orthoester cryptates by varying the substituent on the orthoester bridgehead. Five new cryptates could be isolated with substituents ranging from electron donating (e.g., -*n*C_4_H_9_, -CH_2_C_6_H_5_) to aromatic (e.g., -C_6_H_5_) and mildly electron-withdrawing (e.g., -CH_2_Cl, -C≡C-TMS) groups. Cryptates with strongly electron-withdrawing groups (e.g., -CF_3_) could not be prepared, as they presumably destabilize the oxonium ion intermediate involved in exchange by too much.^38^

In the same study, we were also able to prepare a lithium encapsulating cryptate with LiBArF as the metal template. Interestingly, this Li-templated self-assembly required trimethyl orthoformate (R = H) as the starting material (**[Li**^**+**^**⊂*****o*****-(H)**_**2**_**-1.1.1]BArF**^**–**^). That orthoformates are outliers was further underscored by our studies of binding constants. Host–guest (NMR) titrations were typically performed in acetonitrile, which guarantees the full solubility of added salts, but it is important to note that this solvent differs from the one used for self-assembly experiments (CDCl_3_). We found that ***o*****-(H)**_**2**_**-1.1.1** binds Li^+^ about 2 orders of magnitude more strongly than Na^+^ (*K*_a_ = 13000 M^–1^ and 60 M^–1^, respectively).^38^ In contrast, the binding constants that we determined for orthoacetate host ***o*****-Me**_**2**_**-1.1.1** with Li^+^ and Na^+^ were identical within error (*K*_a_ = 1700 M^–1^ and 1300 M^–1^, respectively).^39^ Detailed analyses of torsion angles (R–C–O–M dihedrals, where R = H or Me, M = Li or Na; SCXRD) shed light on this issue. The average torsion angle of the orthoacetate cryptands is 180°, whereas the average torsion angles of the orthoformate cryptands are 148° (M = Li) and 168° (M = Na). The cavity of orthoformate cryptands is therefore significantly smaller than that of orthoacetate cryptands, explaining the differences in ion selectivity.^40^

To study the effect of the substituent on the reactivity and stability of the orthoester motif, we investigated the rates of the exchange reaction for 13 different trimethyl orthoesters by ^1^H NMR spectroscopy. We observed that the differences in reactivity between orthoesters are so vast that both different amounts and different types of acid had to be used in this fundamental study. In summary, we found a well-behaved trend from less reactive, electron-deficient orthoesters (e.g., -CF_3_, -C≡CH) toward more reactive, electron-rich orthoesters (e.g., -CH_3_). Changing the substituent is therefore an excellent way to fine-tune reactivity and Taft’s “polar parameter” predicts the trend reasonably well.^38^

**Figure 3:**
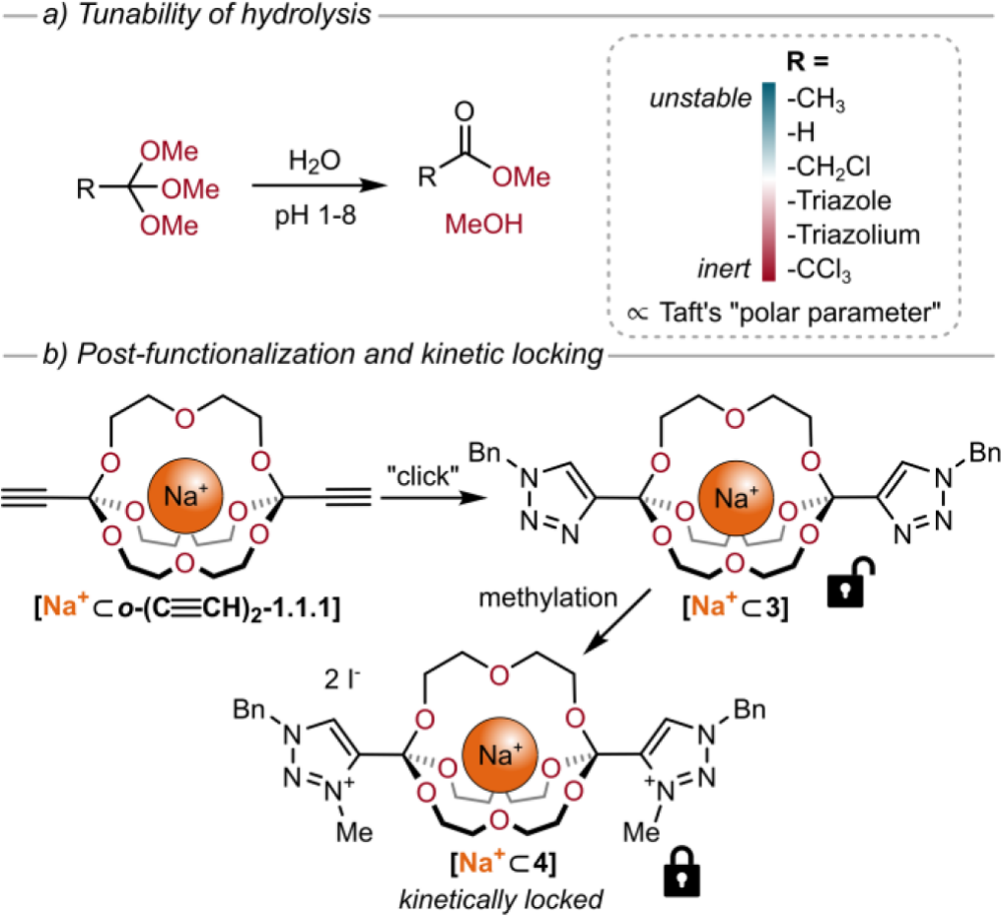
(a) Hydrolysis rates of orthoesters strongly depend on the substituent (R) at the bridgehead. (b) Postfunctionalization and kinetic locking of alkynyl orthoester cryptates.^38^

The same trend of reactivity was observed for the kinetics of hydrolysis. We treated six different orthoesters with phosphate buffers over a broad pH range (1–8) and monitored the degradation reaction by ^1^H NMR spectroscopy. We found that orthoesters equipped with electron-deficient groups can be extremely inert (e.g., *t*_1/2_ = >10000 min at pH 1 for R = -triazolium), while electron-rich groups render orthoesters prone to hydrolyze even under neutral conditions (e.g., *t*_1/2_ = 10 min at pH 7 for R = -CH_3_) (Figure 3a).

To demonstrate that orthoester cryptands are not limited to commercially available orthoester substrates, we prepared the alkyne-substituted cryptate **[Na**^**+**^**⊂*****o*****-(C≡CH)**_**2**_**-1.1.1]BArF**^**–**^ via deprotection from the TMS-substituted precursor cryptate **[Na**^**+**^**⊂*****o*****-(C≡C–Si(CH**_**3**_**)**_**3**_**)**_**2**_**-1.1.1]BArF**^**–**^. This cryptate could be postfunctionalized via Sonogashira reaction and via CuAAC “click” reaction (Figure 3b). By subsequent methylation of the triazole-substituted cryptate **[Na**^**+**^**⊂3]**, positively charged triazolium-variant **[Na**^**+**^**⊂4]** was prepared. Compared to the triazole cryptand **3**, we found that the triazolium cryptand **4** was more stable by at least 4 orders of magnitude, such that hydrolysis was negligible even at pH 1. Triazolium-substituted orthoesters can therefore be considered kinetically locked.^38^ Installation of a stimuli-responsive trigger, that can be cleaved e.g. by esterase,^41^ would allow application of such compounds for the targeted delivery of ions in tissue where esterases are overexpressed and pH is low (e.g., cancer cells).

The kinetic stability of orthoesters can also be increased substantially by substituting oxygen with sulfur.^42^ In 2019, we disclosed a systematic study of the dynamic covalent exchange of trithioorthoesters with thiols in the presence of catalytic amounts of Lewis acids (e.g., FeCl_3_, BF_3_·OEt_2_) or stoichiometric amounts of Brønsted acids (e.g., TFA, triflic acid (TfOH)). While *O*,*O*,*O*-orthoesters degrade already under slightly acidic conditions, *S*,*S*,*S*-orthoesters are less sensitive to moisture as the exchange with thiols can kinetically outcompete hydrolysis.^43^ We believe that trithioorthoester exchange has vast untapped potential^44^ for applications in materials science and will report proof-of-principle studies in due course.

## Adaptive and Dynamic Behavior of Orthoester Cryptates

### Shrinkage and Expansion of the Cavity Size

**Figure 4:**
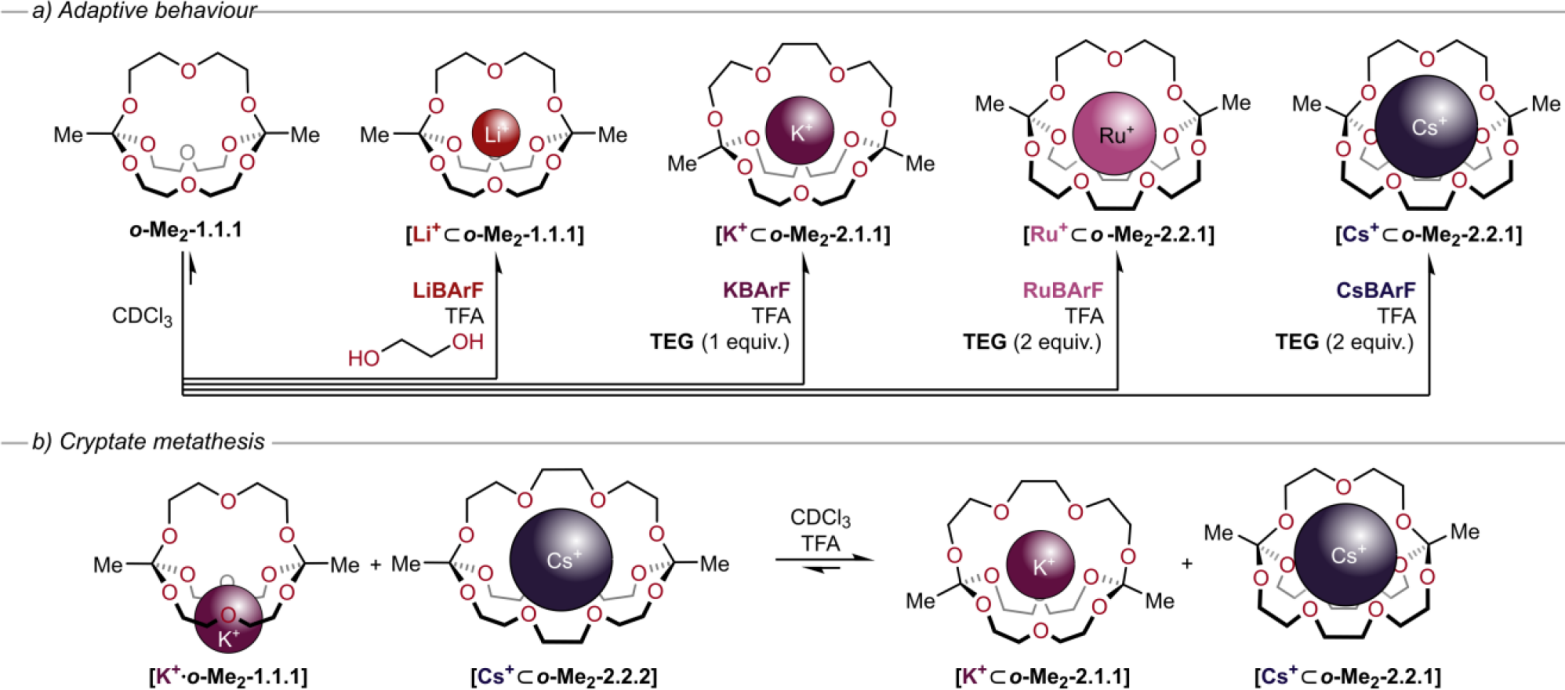
(a) Adaptive behavior of ***o*****-Me**_**2**_**-1.1.1** in the presence of different guest ions and varying amounts of ethylene glycol or triethylene glycol (**TEG**). (b) Cryptate metathesis reaction starting from ***o*****-Me**_**2**_**-1.1.1** and ***o*****-Me**_**2**_**-2.2.2** with mismatching metal ions K^+^ and Cs^+^.^45^

Conventional macrobicyclic hosts exhibit the disadvantage that their framework is no longer dynamic after the completion of their synthesis. Orthoester cryptates, however, are adaptive to their environment under acidic conditions. In 2017, we demonstrated how metal ions of different sizes influence the dynamic mixture and thereby facilitate the self-assembly of their “matching”, i.e. thermodynamically preferred, host.^45^ We tested whether orthoester cryptand ***o*****-Me**_**2**_**-1.1.1** would expand or shrink via subcomponent exchange in the presence of mismatched metal guests and acid catalyst TFA. We found that ***o*****-Me**_**2**_**-1.1.1** did not shrink in response to Li^+^ ions and ethylene glycol either due to an unsuitable size fit for the Li^+^ ion or due to the lack of one oxygen donor atom in the smaller variant of the host. We were more successful, when we investigated the response of the cryptands to the addition of the larger metal ions K^+^, Rb^+^, and Cs^+^, and the “longer” subcomponent tri(ethylene glycol) (**TEG**).^45^ In the presence of potassium, cryptand ***o*****-Me**_**2**_**-2.1.1** formed as the predominant product, whereas cryptand ***o*****-Me**_**2**_**-2.2.1** formed with both rubidium and cesium (Figure 4a).^45^

While it is remarkable that the symmetric templates K^+^, Rb^+^, and Cs^+^ can facilitate a desymmetrization reaction, the route from symmetric to unsymmetric cryptands is rather tedious. We therefore demonstrated that unsymmetric cryptates can also be synthesized via direct self-assembly under standard conditions,^45^ and we note that conventional cryptates of similar complexity require laborious multistep syntheses.^46^ We were also able to perform an elegant “cryptate metathesis” reaction by combining the symmetric cryptands ***o*****-Me**_**2**_**-1.1.1** and ***o*****-Me**_**2**_**-2.2.2** with mismatched metal ions K^+^ and Cs^+^. Addition of TFA initiated a complex subcomponent exchange reaction that gave rise to a 1:1 mixture of the asymmetric cryptates **[K**^**+**^**⊂*****o*****-Me**_**2**_**-2.1.1]** and **[Cs**^**+**^**⊂*****o*****-Me**_**2**_**-2.2.1]** (Figure 4b).^45^

### Inversion of the Bridgehead

**Figure 5:**
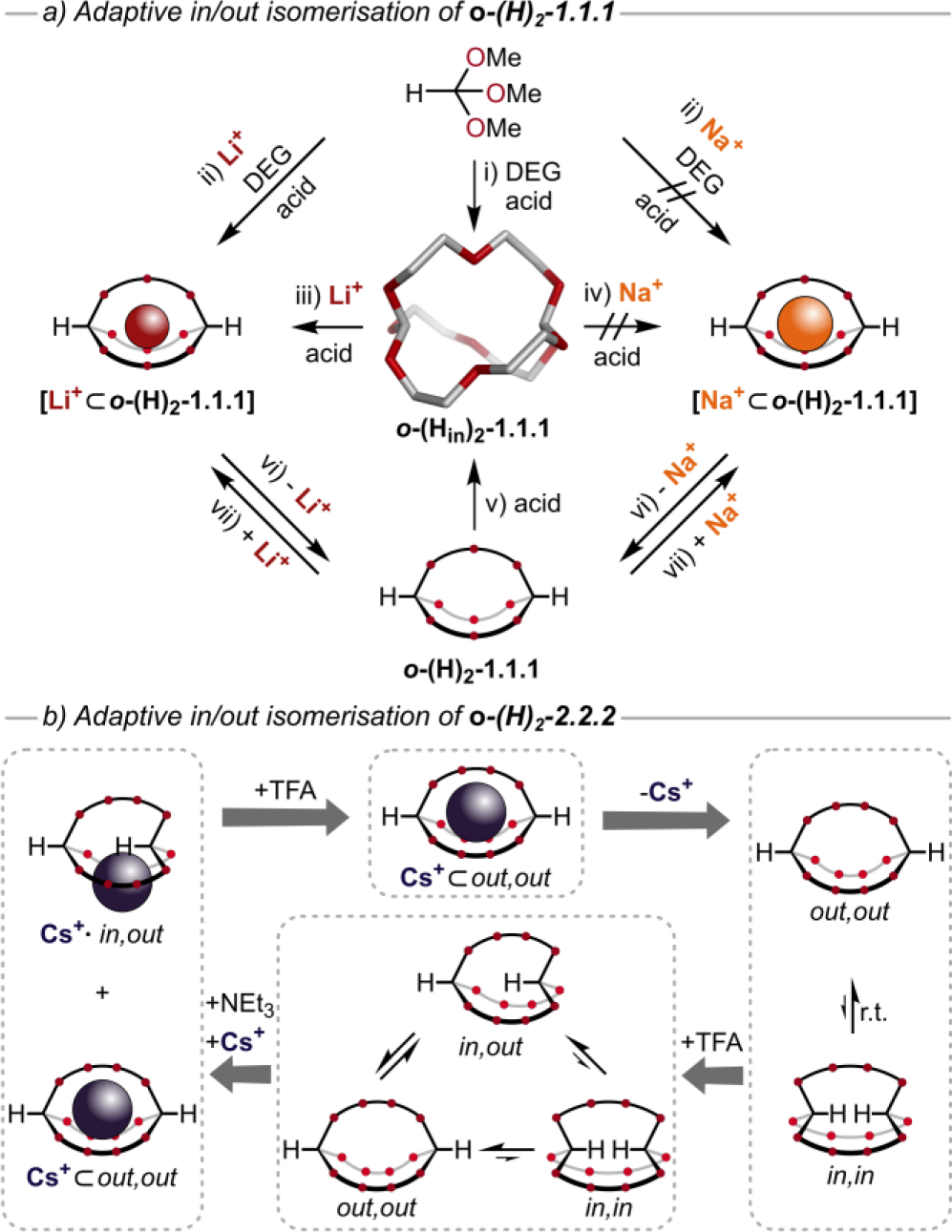
*In*/*out* inversion of orthoformate bridgeheads. (a) ***o*****-H**_**2**_**-1.1.1**(40) with single crystal X-ray structure; (b) ***o*****-H**_**2**_**-2.2.2**.^48^

The size of the cryptand cavity can be tuned not only by exchange of the bridging moieties but also by inversion of the bridgehead. In 2019, we reported on a remarkable feature of the orthoformate bridgehead (R = H): in the absence of any template we were able to isolate the self-templated *in*,*in*-cryptand ***o*****-(H**_**in**_**)**_**2**_**-1.1.1** in 51% yield (Figure 5a). Favorable intramolecular hydrogen bonds between the central oxygen atoms in the **DEG** chain and the hydrogen atoms of the bridgehead, confirmed by SCXRD, provided the thermodynamic driving force for the formation of this exceptionally small cryptand.^40^

With the self-templated *in*,*in*-cryptand ***o*****-(H**_**in**_**)**_**2**_**-1.1.1** and the metal-templated *out*,*out*-cryptand ***o*****-(H**_**out**_**)**_**2**_**-1.1.1** in hand, we set out to study the inversion of the bridgehead using acid-catalyzed dynamic covalent exchange. Indeed, we were able to transform ***o*****-(H**_**out**_**)**_**2**_**-1.1.1** into ***o*****-(H**_**in**_**)**_**2**_**-1.1.1** upon the addition of catalytic amounts of acid. The presence of lithium ions and acid led to the formation of **[Li**^**+**^**⊂*****o*****-(H)**_**2**_**-1.1.1]**, while the subsequent removal of the lithium ions by ion-exchange resin gave back ***o*****-(H**_**out**_**)**_**2**_**-1.1.1** (Figure 5a). To rule out (thermal) homeomorphic isomerization in which inversion of the bridgehead occurs by pulling one chain through the residual macrocycle, ***o*****-(H**_**in**_**)**_**2**_**-1.1.1** was heated for several hours at 200 °C (neat). No inversion was observed, which indicates that this small cryptand requires constitutionally dynamic exchange to “turn itself inside out”.^40^ While the direct synthesis of **[Na**^**+**^**⊂*****o*****-(H)**_**2**_**-1.1.1]** was not possible due to the size difference between orthoformate cryptands and their orthoacetate analogues (see further above), addition of NaBArF to ***o*****-(H**_**out**_**)**_**2**_**-1.1.1** allowed isolation of the sodium cryptate via detour (Figure 5a).^40^

Inspired by studies on the homeomorphic isomerization in large macro(bi)cycles,^47^ we investigated, if this inversion pathway was possible in larger orthoester cages. We therefore synthesized the orthoformate cryptate **[Cs**^**+**^**⊂*****o*****-(H)**_**2**_**-2.2.2]** using our previously optimized conditions^1^ in 95% yield. We found that this compound equilibrates between *out*,*out*-, *in*,*in*-, and *in*,*out*-configurations by homeomorphic isomerization and/or dynamic orthoester exchange (Figure 5b).^48^ When we removed the template after synthesis, we did not end up with a single host but rather a mixture of two isomers. ^1^H NMR analysis revealed the simultaneous formation of stereoisomers ***o*****-(H**_**out**_**)**_**2**_**-2.2.2** and ***o*****-(H**_**in**_**)**_**2**_**-2.2.2** in a 2:1 mixture (in CD_3_CN), which can only be rationalized by homeomorphic isomerization of ***o*****-(H**_**out**_**)**_**2**_**-2.2.2**.

Addition of acid catalyst to initiate dynamic covalent exchange gave rise to the third possible isomer: ***o*****-(H**_**in**_**H**_**out**_**)-2.2.2**. Under these conditions, both dynamic covalent exchange and homeomorphic inversion are possible; hence, the observed mixture of three isomers corresponds to the global thermodynamic equilibrium of the reaction network (molar ratio of *out*,*out*-, *in*,*in*-, and *in*,*out*-cryptand ca. 2:1:2 in CD_3_CN). Addition of base (e.g., Et_3_N) and cesium ions led to the complete disappearance of the *in*,*in*-isomer by homeomorphic isomerization. Subsequent addition of acid to this mixture allowed the *in*,*out*-*exo* complex **[Cs**^**+**^**·*****o*****-(H**_**in**_**H**_**out**_**)-2.2.2]** to turn into the more stable *out*,*out*-*endo* cryptate by orthoester exchange. The reaction scheme shown in Figure 5b therefore represents a “full circle” of homeomorphic and dynamic covalent bridgehead inversions that are driven by the removal or addition of the Cs^+^ template.^48^

### Inherently Dynamic Cryptates: Supramolecular Fluxionality

All members of a well-behaved dynamic combinatorial library are connected through a network of rapid equilibria, where the exchange of subcomponents occurs continuously. As these processes are generally inter- and not intramolecular, they do not meet the definition of fluxionality. In 2019, we showed that some orthoester cryptates exhibit intramolecular constitutional dynamics that allows considering these species as “fluxional host–guest systems”.^2^

We envisaged that the ammonium cation (NH_4_^+^) could be a suitable template for the self-assembly of orthoester cryptates due to its ability to donate strong hydrogen bonds. NH_4_BArF and additional building blocks (**TEG**/**DEG** and trimethyl orthoacetate) were therefore subjected to standard reaction conditions, and we could indeed observe the formation of the corresponding ammonium complex by NMR spectroscopy and ESI mass spectrometry.^2^ However, despite extensive efforts, we were not able to isolate the cryptate. This frustrating finding raised the intriguing question of whether the ammonium guest is sufficiently acidic that it renders orthoester cryptates inherently dynamic, which represents an obvious problem for any aqueous workup procedures.

**Figure 6:**
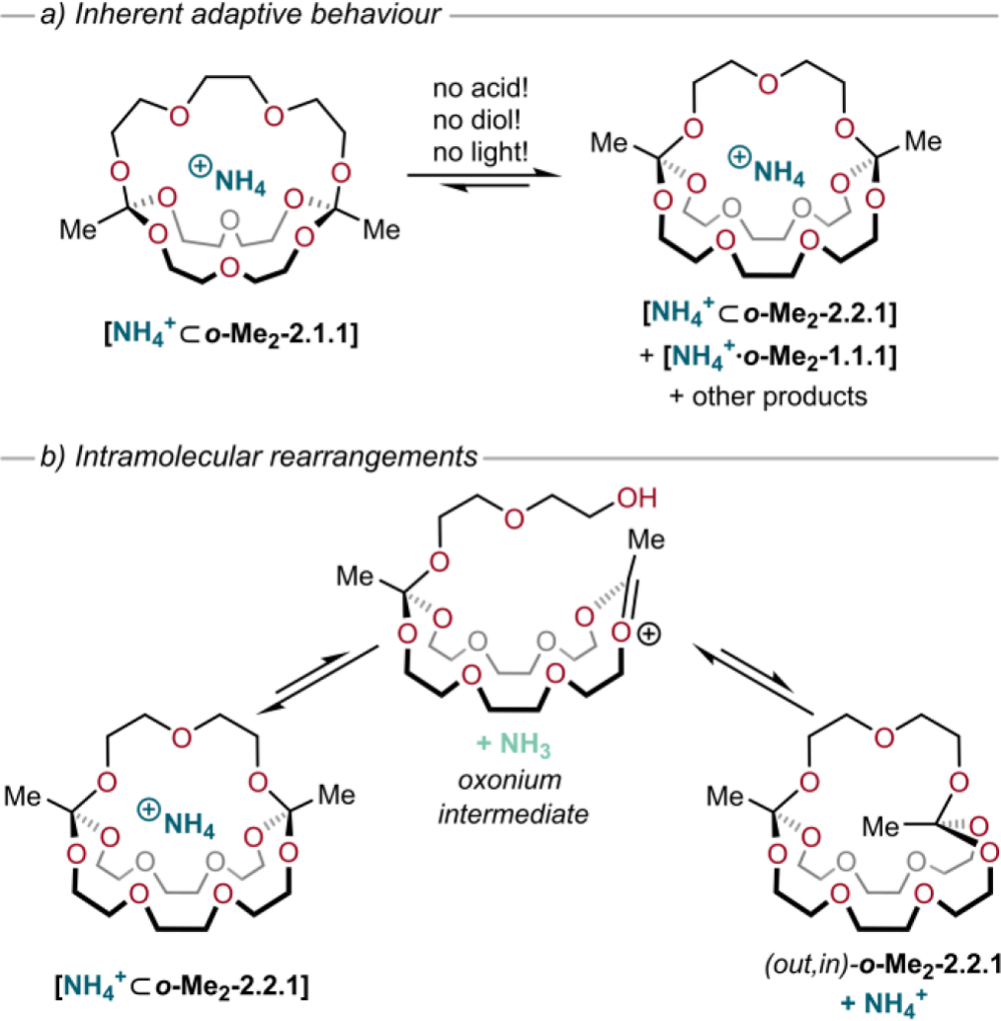
(a) Inherently adaptive behavior of orthoester ammonium cryptates in the absence of acid catalyst, diol, or light. (b) Mechanism of intramolecular rearrangements via proton transfer of the ammonium guest ion to the orthoester carbon, hence forming an oxonium intermediate.^2^

To study the constitutional dynamics in such host–guest systems in detail, we monitored the ammonium complex of cryptand ***o*****-Me**_**2**_**-2.1.1** under careful exclusion of light and in the absence of any free diols or acid. We found that over 48 h, cryptate **[NH**_**4**_^**+**^**⊂*****o*****-Me**_**2**_**-2.1.1]** converted into a mixture with the larger cryptate **[NH**_**4**_^**+**^**⊂*****o*****-Me**_**2**_**-2.2.1]** (ratio 2.5:1 in favor of the larger cage) (Figure 6a). This finding indicates that the larger cryptate is thermodynamically more stable than **[NH**_**4**_^**+**^**⊂*****o*****-Me**_**2**_**-2.1.1]**. Interestingly, the system also responded by forming the *exo* complex **[NH**_**4**_^**+**^**·*****o*****-Me**_**2**_**-1.1.1],** not because this complex is particularly stable, but because the excess of the “short” diol **DEG** has to be accommodated (the ***o*****-Me**_**2**_**-1.1.1** cryptand is an agonist to the true thermodynamic minimum: ***o*****-Me**_**2**_**-2.2.1**).^2^

We hypothesized that the spontaneous rearrangement described above proceeds via free diols generated by small amounts of hydrolysis.^2^ To probe this mechanistic scenario, we added **DEG** or **TEG** to pristine **[NH**_**4**_^**+**^**·*****o*****-Me**_**2**_**-1.1.1]**, **[NH**_**4**_^**+**^**⊂*****o*****-Me**_**2**_**-2.1.1]**, or **[NH**_**4**_^**+**^**⊂*****o*****-Me**_**2**_**-2.2.1]**. After only 4 h, equilibrium was reached, and **[NH**_**4**_^**+**^**⊂*****o*****-Me**_**2**_**-2.2.1]** was formed as the main product in all three experiments, indicating that free diols are involved in this type of subcomponent exchange reaction. Additionally, ^1^H NMR titrations were performed to better understand the thermodynamics of these host–guest systems. As expected, the highest affinity toward NH_4_^+^ was observed for ***o*****-Me**_**2**_**-2.2.1** (*K*_a_ = around 6000 M^–1^ in CD_3_CN) compared to cryptands ***o*****-Me**_**2**_**-1.1.1**, ***o*****-Me**_**2**_**-2.1.1**, and ***o*****-Me**_**2**_**-2.2.2** (*K*_a_ = around 540, 1100, and 400 M^–1^, respectively).^2^

Molecular dynamics (MD) simulations were performed by Christof Jäger to complement these experiments and gain insights that are beyond experimentation. These studies confirmed that ***o*****-Me**_**2**_**-1.1.1** is too small to encapsulate the NH_4_^+^ ion, resulting in *exo* binding, whereas ***o*****-Me**_**2**_**-2.2.2** is too large, leading to a highly dynamic *endo* mode of binding.^2^ Quantum mechanical and molecular mechanics (QM/MM) simulations were performed to study the proton transfer between the cryptand and ammonium guest ion (Figure 6b). The simulations revealed a ring-opening transition state that represents a concerted mechanism with simultaneous C–O bond dissociation and proton transfer, resulting in the formation of the oxonium ion. This mechanism is very slow due to a high kinetic barrier of 16.2 kcal mol^–1^ (related concept: “complexation-induced p*K*_a_ shift”), thus the effective concentration of oxonium ions is low. According to these QM/MM simulations, the formed NH_3_ tends to leave the open host immediately after oxonium ion formation.^2^

After formation of the oxonium intermediate and release of NH_3_ into the solvent, the alcohol group can attack the electrophilic carbon atom from the top (leading back to the starting material) or swing around and attack from the bottom, thus leading to inversion of the absolute configuration of the bridgehead and formation of the *out*,*in*-isomer (Figure 6b). As the (*out*,*in*)-***o*-Me**_**2**_**-2.1.1** cryptand is unable to encapsulate the ammonium ion, there is no “complexation-induced p*K*_a_ shift”; thus attack by an external ammonium ion and formation of the (*out*,*out*) cryptate will be both fast and thermodynamically favored. Based on these simulations and the observation of inherently dynamic rearrangements (Figure 6a), it seems extremely likely that the processes shown in Figure 6b are indeed occurring, which would mean that the diol subcomponents are constantly swapping their place and the host–guest complex can be considered fluxional.

In the context of the literature on supramolecular enzyme mimics, our ammonium cryptates represent a “perfect opposite” of Bergman and Raymond’s self-assembled cage with an orthoformate guest that famously allowed “acid catalysis in basic solution”.^49^ While in Raymond’s case the reactivity of the orthoester guest is modulated by the anionic cage, in our case it is the reactivity of the orthoester cage that is modulated by the ammonium guest. Complex **[NH**_**4**_^**+**^**⊂*****o*****-Me**_**2**_**-2.2.1]** is also an excellent example for teaching the difference between thermodynamic and kinetic stabilities to undergraduate students. The compound is thermodynamically stable, and as such there are no discernible changes in the NMR spectrum over a month, but it is far from kinetically stable, as it turns itself constantly inside out via *intra*molecular rearrangements (and it will also react *inter*molecularly within hours, if additional diol is added).^2^

## Self-Assembly of Anion- and Ion-Pair-Templated Orthoester Cryptates

Orthoester cryptands hydrolyze with tunable rate constants^38^ and offer therefore a pH-driven release mechanism. This stimuli-responsive release mechanism would be of particular interest for the binding and release of anions (supramolecular medicinal chemistry).^50,51^

**Figure 7:**
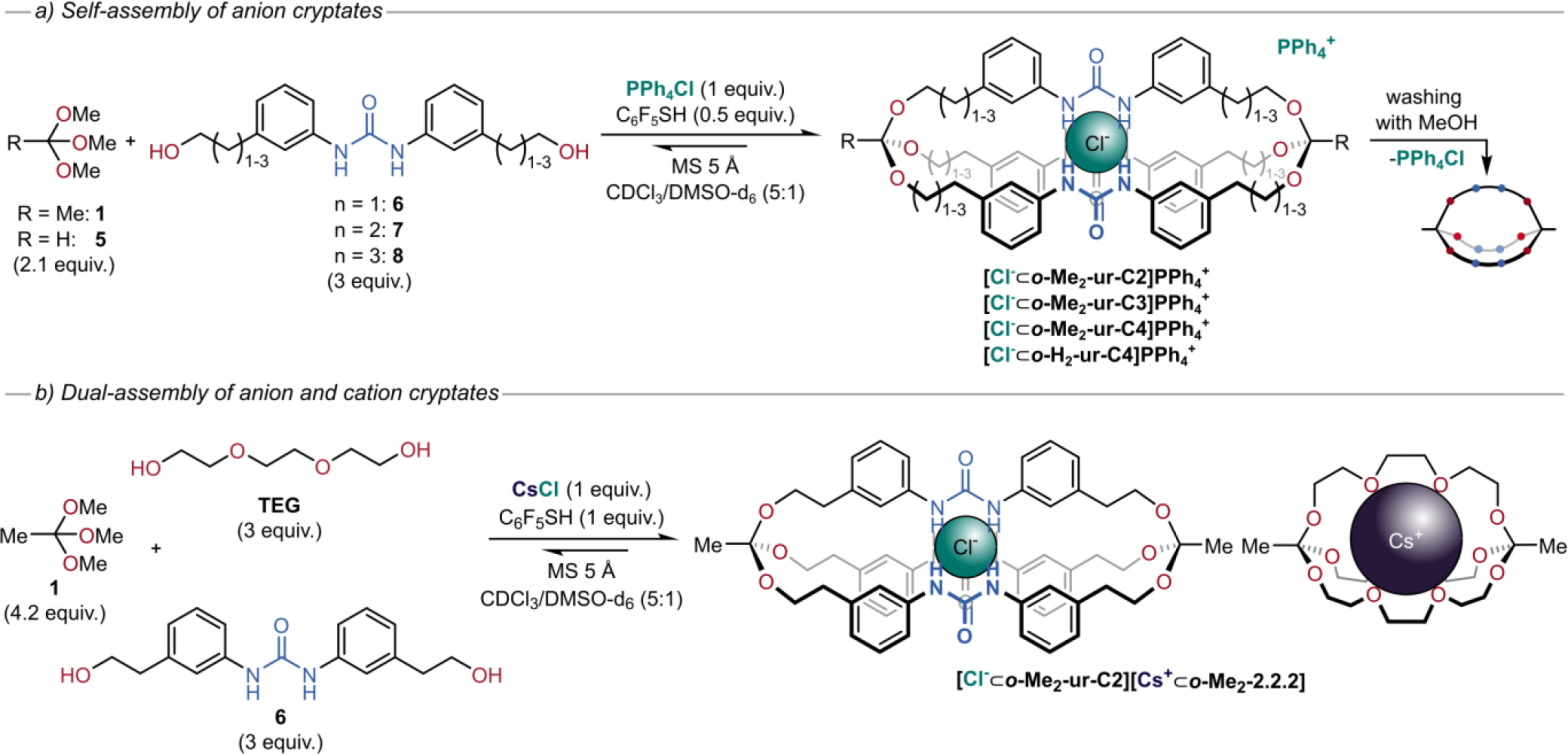
(a) Self-assembly of anion-templated orthoester cryptates with bishydroxyalkylphenyl urea ligands and subsequent removal of the chloride template. (b) Dual-assembly of both anion and cation cryptates using cesium chloride as “ion-pair template”.^3^

To self-assemble anion-binding orthoester cryptates, we designed bishydroxyalkylphenyl urea ligands with varying chain lengths (C_2_ to C_4_) to act as ligands for anion binding and to exchange with the orthoester bridgehead. Our standard acid catalyst TFA could not be used in the self-assembly experiments, as its conjugate base would compete with the anion of interest. We therefore chose 2,3,4,5,6-pentafluoro-thiophenol, whose conjugate thiolate would bind to urea only very weakly. To ensure solubility of all subcomponents, we were restricted to a solvent mixture of chloroform and DMSO (5:1). Tetraphenylphosphonium chloride was added to provide a chloride template and trimethyl orthoacetate or orthoformate were used as the orthoester bridgehead. After a few hours (3–18 h), equilibrium was reached with significant amounts of the cryptates formed. Three different orthoacetate cryptates (**[Cl**^**–**^**⊂*****o*****-Me**_**2**_**-ur-C2]**, **[Cl**^**–**^**⊂*****o*****-Me**_**2**_**-ur-C3]**, and **[Cl**^**–**^**⊂*****o*****-Me**_**2**_**-ur-C4]**) and one orthoformate cryptate (**[Cl**^**–**^**⊂*****o*****-H**_**2**_**-ur-C4]**) were successfully isolated and characterized (Figure 7a).^3^

MD simulations by Christof Jäger showed that DMSO forms hydrogen bonds with the urea moieties, whereas an intramolecular hydrogen bond network between urea groups is present in chloroform. When the chloride ion is present, all three urea functionalities complex the ion in the predominant structure in both solvents. In DMSO and in the solvent mixture used in the self-assembly reactions (chloroform/DMSO 5:1), there exists an additional binding mode where one solvent molecule enters the cavity and occupies one urea entity, leaving only two urea groups for chloride binding.^3^

The chloride template could be removed by treating the cryptates with anhydrous methanol to selectively dissolve tetraphenylphosphonium chloride. With the empty cryptands in hand, we performed ^1^H NMR titrations to determine the binding constants to different anions (Cl^–^, Br^–^, I^–^, and NO_3_^–^). For solubility reasons, most titration were performed in DMSO, which is a highly competitive solvent due to its high dielectric constant (ε = 47^52^) and highly Lewis basic oxygen atom. For this reason, all binding constants were relatively low (highest *K*_a_ = 37 M^–1^ for Cl^–^ to ***o*****-Me**_**2**_**-ur-C2**).^3^ It is worth noting that binding affinities in this moderate range can be advantageous for applications in membrane transport.^53^

To increase the architectural complexity and identify the limit for Cl^–^ encapsulation, we also self-assembled heteroleptic cryptates using mixtures of diols with different chain lengths. The smallest cage we obtained was **[Cl**^**–**^**⊂*****o*****-Me**_**2**_**-ur-(C1)**_**2**_**C2]** which contained two **C1** diols and one longer **C2** diol. We were able to investigate the stability of such heteroleptic cryptands and the corresponding chloride complexes by using tandem mass spectrometry (ESI-MS/MS). Conveniently, this technique allowed us to study the cryptates without the need to isolate each compound.^3^

In unprecedented experiments, we were able to use the salt CsCl as an “ion pair template” for the simultaneous self-assembly of suitable hosts for both the cation and the anion. Therefore, we used **TEG** as the ligand for the cesium ion, a bishydroxyalkylphenyl urea diol (**6**) as the ligand for the chloride ion and CsCl to provide both templates simultaneously (Figure 7b). The structure of **[Cl**^**–**^**⊂*****o*****-Me**_**2**_**-ur-C2][Cs**^**+**^**⊂*****o*****-Me**_**2**_**-2.2.2]** was confirmed by ^1^H NMR spectroscopy, attenuated total reflection-infrared (ATR-IR) spectroscopy, and high resolution ESI-MS. The association of both hosts to both guest ions was confirmed by ^1^H and ^133^Cs NMR spectroscopy.^3^

To shed light on the driving force behind the formation of each cryptate, we performed competitive experiments with under-stoichiometric amounts of trimethyl orthoacetate (2.1 equiv). While in the standard experiment both cryptates were obtained in ca. 1:1 ratio, in the competitive experiment, **[Cl**^**–**^**⊂*****o*****-Me**_**2**_**-ur-C2]** and **[Cs**^**+**^**⊂*****o*****-Me**_**2**_**-2.2.2]** formed in ca. 2:1 ratio, indicating a significant preference of the system for chloride rather than cesium cryptate formation. To follow up on this unexpected finding, we attempted to self-assemble the cryptates separately using CsCl as a template. While **[Cl**^**–**^**⊂*****o*****-Me**_**2**_**-ur-C2]Cs**^**+**^ was obtained in 42% yield, only traces of **[Cs**^**+**^**⊂*****o*****-Me**_**2**_**-2.2.2]Cl**^**–**^ formed. We therefore conclude that the chloride cryptate provides most of the driving force in the dual-assembly reaction and the cesium cryptate only forms as a consequence of the formation of **[Cl**^**–**^**⊂*****o*****-Me**_**2**_**-ur-C2]** (another case of an agonistic relationship).^3^ Our dual-assembly reaction (in chloroform/DMSO 5:1) therefore represents a rare example where the binding of Cl^–^ both outcompetes and enables the binding of Cs^+^. A related “modular” self-assembly concept has recently been utilized by Flood and co-workers.^54^

## Trialkoxysilane Exchange, Cryptates, and pH-Divergent Hydrolysis

The dynamic Si–O bond was recently utilized to prepare vitrimers, thermosets, and macrocycles.^55−57^ Having noticed that the DCvC of trialkoxysilanes remained unexplored, we set out to tackle the difficult challenge of identifying mild conditions for this typically very harsh exchange reaction.

**Figure 8:**
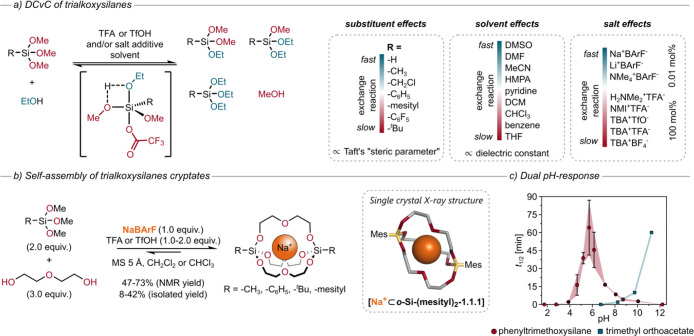
(a) Substituent, solvent, and salt additive effects on trialkoxysilane exchange. (b) Self-assembly of trialkoxysilane cryptates. Representative single crystal X-ray structure of **[Na**^**+**^**⊂*****o*****-Si-(mesityl)**_**2**_**-1.1.1]** (Mes = mesityl; omitted for clarity). (c) Half-lives *t*_1/2_ for the hydrolysis of phenyltrimethoxysilane (red) and trimethyl orthoacetate (blue).^4^

We studied the reaction of seven different trimethoxysilanes with ethanol in chloroform in the presence of an acid catalyst (Figure 8a). It became evident that the reactivity of the trialkoxysilanes strongly depends on the substituent at the Si bridgehead. With increasing steric demand of the substituent, higher amounts of acid and stronger acid catalysts were necessary to bring the exchange reactions to equilibrium within reasonable time. While only 10 mol % of TFA was sufficient to equilibrate trimethoxysilane (R = H) in less than 1 h, 50 mol % of TfOH was necessary for the *t*-butyl analogue to equilibrate within ca. 3.5 h. The reactivity of the trialkoxysilanes correlated roughly with Taft’s “steric parameter”, whereas orthoester reactivity correlated with Taft’s “polar parameter”. The difference stems from the mechanism of the exchange reaction, which in the case of trialkoxysilanes features a penta-coordinated intermediate, where sterics play a more important role than electronics (in contrast to orthoester exchange). DFT calculations carried out by Lutz Greb revealed a specific effect of the TFA acid catalyst, namely, that it generates a penta-coordinated adduct (Figure 8a), which lowers the barrier for ethanol exchange.^4^

To study the influence of the solvent on the reaction kinetics, we chose seven aprotic solvents spanning a vast range of dielectric constants as well as two basic solvents (hexamethylphosphoric triamide (HMPA) and pyridine). The kinetic data obtained by ^1^H NMR spectroscopy revealed a linear dependence of the solvent polarity on the reaction rate.

For instance, a conversion of only 3% was found in benzene after 20 min, whereas in DMSO the equilibrium was reached after only 7 min.^4^

The basic solvents pyridine and HMPA represented outliers: reactivity was higher than expected from their dielectric constants. Because these solvents form salts with the acid catalyst TFA, we proceeded to study the influence of salt additives on the reaction kinetics (trifluoroacetate salts, triflate salts, and various salts comprising weakly coordinating ions). The experimental data revealed a rate enhancing effect of the trifluoroacetate and triflate salts, and two of these salts even facilitated the exchange reaction in the absence of acid. The combination of small, coordinating cations with weakly coordinating anions (NaBArF, LiBArF, and NMe_4_BArF) also gave rise to remarkably mild exchange conditions. Only 0.1 mol % of these salts was enough to significantly enhance the reaction rate. Computational studies confirmed that the trifluoroacetate anion has a barrier-lowering effect and that general electrostatic effects (e.g., external electric fields) also facilitate the exchange reaction.

Encouraged by these findings, we explored the synthesis of trialkoxysilane cryptates (Figure 8b). For a typical self-assembly reaction, we used a trimethoxysilane, **DEG** as a ligand for metal-binding, TFA or TfOH as acid catalyst, chloroform or DCM as solvent, and MS 5 Å. NaBArF was added, this time not only to provide a metal template but also to enhance the reaction rate. To our delight, we were able to isolate four trialkoxysilane cryptates and even obtained two single crystal X-ray structures that revealed pronounced structural differences between trialkoxysilane and orthoester bridgeheads (compare Figure 8b with Figure 2).

The isolation of trialkoxysilane cryptates was more challenging than expected, especially when compared with their orthoester analogues, which we often simply passed through a short plug of silica gel. Hydrolysis studies at varying pH showed that the reason for these difficulties is that trialkoxysilanes hydrolyze under both acid and basic conditions (Figure 8c). Isolating orthoesters is therefore relatively straightforward, as long as any sources of acid are avoided, whereas the isolation of trialkoxysilanes is like balancing on a knife’s edge, because both acid and base can lead to undesired hydrolysis. This divergent pH-response is a practical challenge during synthesis, but it provides unique opportunities for applications of small-molecule and polymeric products, for instance, in systems chemistry and drug delivery.

## Concluding Remarks

**Figure 9:**
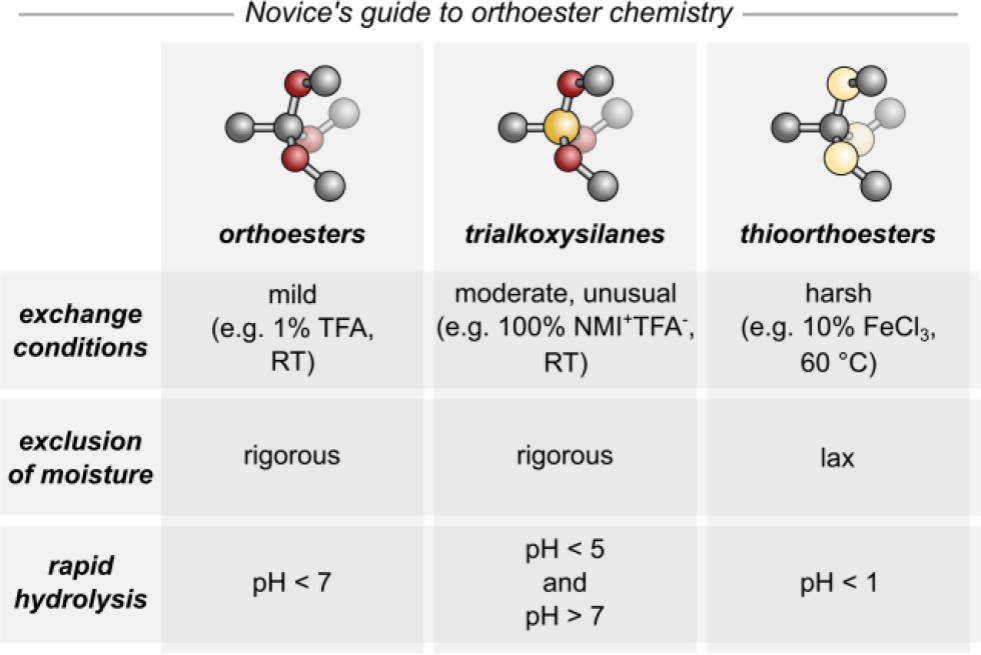
General guidance on conditions for exchange and hydrolysis (NMI = *N*-methylimidazolium; for data on specific R substituents please refer to ref (33) (orthoesters), ref (4) (trialkoxysilanes), and ref (43) (thioorthoesters)).

Over the past decade, we have explored the dynamic covalent exchange of tripodal functional groups: *O*,*O*,*O*-orthoesters, *S*,*S*,*S*-trithioorthoesters, and trialkoxysilanes. We have investigated fundamental aspects including reactivity, hydrolytic stability, and reaction mechanisms, and we have used these reactions to create self-assembled supramolecular hosts for the encapsulation of simple cations and anions. Thanks to their dynamic bridgeheads, these compounds are adaptive and degradable in a highly pH-dependent and predictable fashion. We hope that this Account will inspire further uses of these inherently tripodal dynamic covalent motifs and therefore disclose a “novice’s guide” to the chemistry of *O*,*O*,*O*-orthoesters, *S*,*S*,*S*-trithioorthoesters, and trialkoxysilanes in Figure 9. Future work in our laboratory will increasingly focus on molecules and materials enabling controlled delivery and release.
